# Blood transcriptomic immune signatures in herpes zoster and postherpetic neuralgia: parallel cohort analyses of IFN-related pathway patterns

**DOI:** 10.3389/fcimb.2026.1926554

**Published:** 2026-09-23

**Authors:** Huiying Zhang, Yidong Zhu, Weijie Shao, Kai Xu

**Affiliations:** 1 Department of Pain Medicine, Shaoxing People’s Hospital, Shaoxing, China; 2 Hangzhou Sun Taihe Traditional Chinese Medicine and Pharmacy, Hangzhou, China; 3 Hangzhou Fuyang Hospital of TCM Orthopedics and Traumatology, Hangzhou, China; 4 The Second Affiliated Hospital of Zhejiang Chinese Medical University, Hangzhou, China

**Keywords:** bulk RNA-seq, druggable axis, herpes zoster, immune deconvolution, postherpetic neuralgia, type I interferon, varicella-zoster virus, WGCNA

## Abstract

Herpes zoster (HZ) results from reactivation of latent varicella-zoster virus (VZV), and postherpetic neuralgia (PHN) is a major chronic pain complication. We performed parallel analyses of two independent public blood transcriptomic cohorts: GSE242252 (n=80; HZ onset, 1-year recovery, and controls) and PRJNA1142765 (n=23; 15 HZ and 8 PHN). Because the cohorts differed in platform, library preparation, design, and sampling structure, expression matrices were not merged; cross-cohort interpretation was restricted to within-cohort results and pathway-level directional concordance. HZ onset showed strong type I interferon (IFN)-dominated activation (HALLMARK_INTERFERON_ALPHA_RESPONSE, NES = 2.21, adjusted p=4.4x10-10), with recovery-associated attenuation of the sample-level IFN score (onset vs. recovery, p=0.005). In the smaller PHN cohort, only two genes met the strict differential-expression threshold, but Hallmark GSEA showed positive IFN-alpha and IFN-gamma enrichment. Ranking-sensitivity analysis confirmed IFN-alpha and IFN-gamma as the top two positively enriched Hallmark pathways using either log2 fold change or the DESeq2 Wald statistic, with leading-edge Jaccard similarities of 0.848 and 0.897, respectively. Exact patient-label permutation of the sample-level IFN score was not significant (two-sided p=0.297), although the PHN-minus-HZ direction remained positive in all 23 leave-one-out iterations. The new biological information is therefore an asymmetry of evidence: acute HZ IFN activation and recovery-associated attenuation are strongly supported, whereas the PHN-associated IFN pattern is stable at the pathway-ranking level but statistically uncertain at the patient level. Accordingly, the prior interpretation of persistent IFN activation in PHN is narrowed rather than reinforced. A structured external-replication search did not identify a suitable independent public human blood/PBMC transcriptomic cohort containing PHN samples; external evidence was therefore treated as literature-based concordance and supportive context rather than validation. Restricted computational prioritization highlighted IFN-related and CCL5/CCR5-associated axes for future study.

## 1 Introduction

Herpes zoster (HZ), commonly known as shingles, is caused by the reactivation of latent varicella-zoster virus (VZV) in sensory ganglia. The global incidence of HZ is approximately 3–5 cases per 1,000 person-years, increasing significantly with age due to immunosenescence (Curran et al., 2021; Patil et al., 2022). While acute HZ is typically self-limiting, postherpetic neuralgia (PHN) - pain persisting for more than 3 months after rash healing - develops in 10-30% of HZ patients and represents the most common and debilitating complication (Li et al., 2024; Liu et al., 2024).

The immunobiology of HZ and PHN spans systemic antiviral immunity and disease processes within peripheral sensory tissues. VZV-specific CD4+ and CD8+ T-cell responses are important for controlling viral reactivation (Laing et al., 2018; Peng et al., 2022), while type I and type II interferon pathways provide distinct antiviral defense layers (Sen et al., 2018; Gerada et al., 2020). However, peripheral-blood transcriptomics measures a systemic immune compartment and cannot directly resolve molecular events in dorsal root ganglia, sensory neurons, satellite glial cells, or other affected neural tissues. Accordingly, blood-based observations should be interpreted as peripheral immune correlates rather than direct evidence of PHN neural pathogenesis.

Recent studies have begun to characterize blood and immune-cell signatures of HZ and PHN. Single-cell profiling of PBMCs has described immune changes during HZ onset and recovery (Zheng et al., 2024), while Wang et al. reported whole-blood transcriptomic differences between HZ and PHN, including type I IFN-related and neutrophil-associated signals (Wang et al., 2024). Additional HZ studies have characterized HLA-associated immune signatures and TCR–epitope functional responses (Boeren et al., 2024; Vandoren et al., 2024). CyTOF analysis has also demonstrated immune-landscape differences across HZ and PHN (Peng et al., 2022). These studies establish systemic immune involvement but leave two questions unresolved: how strongly acute antiviral programs attenuate during recovery, and how much of the PHN-versus-HZ blood signal remains stable when subjected to small-sample robustness testing.

The biological contribution of the present work is therefore framed as an evidence hierarchy rather than as the addition of computational tools. The new information is the asymmetry of evidence across disease contexts: the independent GSE242252 cohort provides robust within-cohort evidence for acute HZ IFN activation with recovery-associated attenuation, whereas PRJNA1142765 provides a separate cross-sectional PHN-versus-HZ comparison in which the IFN pattern remains stable at the pathway-ranking level but statistically uncertain at the patient level. We test whether the PHN-associated IFN ranking survives alternative gene-ranking definitions and sample deletion, while separately evaluating patient-label permutation at the sample-score level. This evidence asymmetry narrows, rather than reinforces, the previous interpretation of persistent IFN activation in PHN and explicitly avoids inferring temporal persistence, neural mechanisms, or validated therapeutic targets from the available data.

In this study, we analyzed two independent public blood transcriptomic datasets in parallel to characterize HZ onset/recovery and PHN-versus-HZ immune patterns. The cohorts were not pooled or batch-corrected across studies. Cross-cohort interpretation was limited to directional concordance of pathway- and regulatory-level summaries. We further performed targeted robustness analysis for the PHN IFN finding, summarized available clinical covariates, assessed the feasibility of independent transcriptomic replication, and retained drug-related results only as restricted hypothesis-generating pathway annotations. The study design is summarized in **Figure 1**.

**Figure 1:**
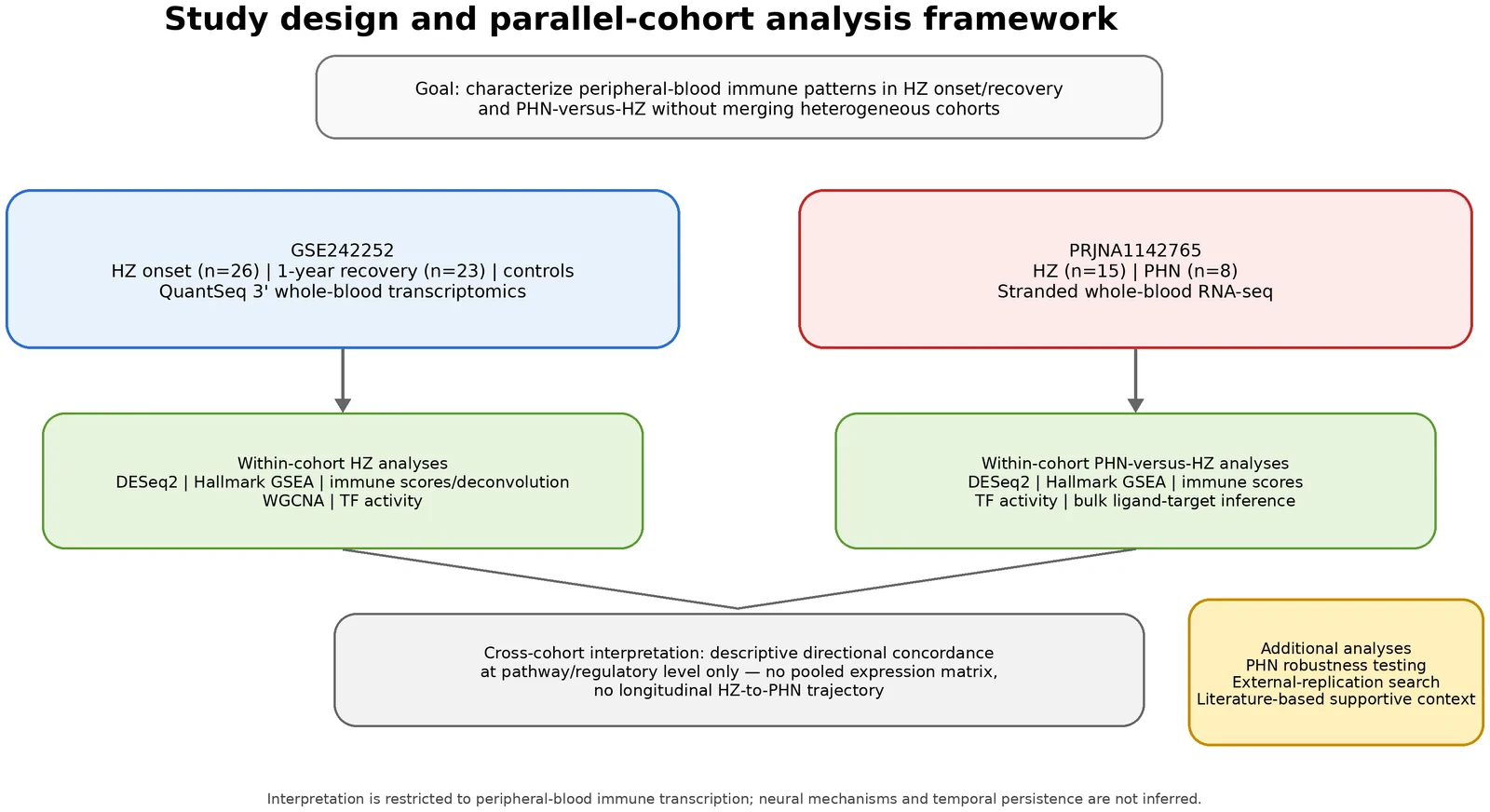
Study design and parallel-cohort analysis framework. GSE242252 and PRJNA1142765 were analyzed as independent cohorts rather than merged into a single expression dataset. Within-cohort modules were performed separately. Cross-cohort interpretation was restricted to descriptive pathway/regulatory directional concordance. Additional analysis included PHN robustness testing and a structured search for an independent PHN transcriptomic replication cohort; supportive literature and external datasets were not labeled as independent validation.

## 2 Materials and methods

### 2.1 Datasets

Two independent public datasets were analyzed. GSE242252 (Gene Expression Omnibus) comprised 80 samples: 26 HZ onset, 23 HZ 1-year recovery, 22 control 1-year, 6 control baseline, and 3 ERC samples, profiled using QuantSeq 3’ mRNA sequencing on the NextSeq 500 platform. PRJNA1142765 (Sequence Read Archive) comprised 23 whole-blood samples (15 HZ and 8 PHN), generated using an Illumina stranded mRNA library and paired-end sequencing on the NovaSeq Xplus platform (Wang et al., 2024). The PRJNA1142765 source study reported age, sex, Visual Analog Scale (VAS), disease course, zoster location, and chronic medical conditions; these variables are summarized in **Table 1**. Detailed antiviral regimens, vaccination status, analgesic exposure, and exact participant-level blood-sampling intervals beyond the reported disease-course summaries were not publicly available.

**Table 1:** Reported clinical and demographic characteristics of the PRJNA1142765 HZ/PHN cohort (Wang et al., 2024).

Characteristic	PHN (n=8)	HZ (n=15)	Source-study p-value
Average age, years	63.875	66.13	0.6788
Average VAS	7.625	7.93	0.6269
Male	4 (50%)	6 (40%)	0.6850
Female	4 (50%)	9 (60%)	–
Face involvement	2 (25%)	1 (6.7%)	0.2688
Neck/upper limbs	2 (25%)	4 (26.7%)	>0.9999
Trunk	4 (50%)	10 (66.7%)	0.6570
Disease course, range	90–365 days	10–60 days	–
Disease course, Q1	90 days	20 days	–
Disease course, median	105 days	23 days	–
Disease course, Q3	232.5 days	40 days	–
Hypertension	4 (50%)	6 (66.7%)	0.6850
Diabetes	1 (12.5%)	1 (6.7%)	>0.9999
Coronary heart disease	1 (12.5%)	2 (13.3%)	>0.9999
Valvular heart disease	1 (12.5%)	0	0.4211
Lung disease	2 (25%)	1 (6.7%)	0.5459
Leukemia	1 (12.5%)	1 (6.7%)	>0.9999
Cerebral infarction	1 (12.5%)	3 (20%)	0.6027
Mild depression	0	1 (6.7%)	>0.9999
Asthma	0	1 (6.7%)	>0.9999
Treatment before chronic pain	All 8 PHN participants reported as having received treatment before chronic pain	Not reported in comparable form	–
Vaccination status	Not reported	Not reported	–
Detailed antiviral regimen	Not reported	Not reported	–

Values are reproduced from the PRJNA1142765 source publication (Wang et al., 2024). The source report stated that age, VAS, sex, zoster location, and chronic-disease distributions did not differ significantly between groups. Formal age matching was not reported. Disease-course summaries reflect different clinical stages and do not provide exact participant-level blood-sampling intervals. Detailed antiviral regimens, vaccination history, and analgesic exposure were unavailable for covariate adjustment in this secondary analysis.

The two cohorts were analyzed independently from preprocessing through statistical testing. Because study identity was inseparable from platform, library preparation, cohort design, processing history, and clinical sampling structure, a global cross-study batch correction or single numerical batch-effect estimate would not distinguish technical from biological effects and was therefore not applied. No expression matrices were merged, and no cross-study meta-analysis or direct effect-size pooling was performed. Cross-cohort statements were restricted to directional concordance of within-cohort pathway, immune-signature, and transcription-factor summaries. A structured comparison of cohort design, sequencing technology, processing, biological contrast, and the corresponding analytical mitigation is provided in **Supplementary Table 5**.

### 2.2 RNA-seq Processing

For GSE242252, per-sample count files were assembled into a gene-by-sample count matrix. Ensembl gene IDs were mapped to gene symbols using org.Hs.eg.db, and genes were filtered to retain those with count >= 10 in at least 10% of samples (18,771 genes retained). For PRJNA1142765, raw FASTQ files were downloaded via fasterq-dump and quantified using Salmon v2.1.2 (Patro et al., 2017) with selective alignment (Srivastava et al., 2020) against the GENCODE v44 transcriptome (k=31, --gencode, --validateMappings, --gcBias). Transcript-level estimates were summarized to gene-level counts using tximport (Soneson et al., 2015).

### 2.3 Differential expression analysis

Differential expression analysis was performed using DESeq2 (Love et al., 2014) with the threshold of |log2 fold change| >= 1 and adjusted p-value < 0.05 (Benjamini-Hochberg correction). For GSE242252, three comparisons were performed: (1) HZ onset vs. all controls, (2) HZ recovery vs. controls, and (3) HZ onset vs. HZ recovery (paired design). For PRJNA1142765, PHN vs. HZ was compared.

### 2.4 Gene set enrichment analysis

Gene Set Enrichment Analysis (GSEA) was performed using fgsea against the MSigDB Hallmark gene-set collection (Liberzon et al., 2015). Primary analysis ranked genes by log2 fold change, with minimum gene-set size 10 and maximum size 500. Over-representation analysis was performed using clusterProfiler (Wu et al., 2021) for GO Biological Process and KEGG pathways.

As a robustness analysis for PRJNA1142765, Hallmark preranked GSEA was repeated using two ranking metrics: log2 fold change and the reconstructed DESeq2 Wald statistic (log2FoldChange/lfcSE). Both analysis used the same 50 Hallmark sets (MSigDB 2026.1.Hs), minSize=10, maxSize=500, weighted enrichment score exponent 1, 20,000 same-sign gene-set permutations with adaptive multilevel tail estimation, and fixed seed 20260810. Leading-edge overlap between ranking strategies was summarized using the Jaccard index. In a complementary sample-level analysis, the mean PHN-minus-HZ IFN score was evaluated by exact enumeration of all 490,314 allocations of 8 PHN labels among 23 samples using a two-sided test. To provide an effect-size interval, a 95% percentile bootstrap confidence interval for the PHN-minus-HZ mean IFN-score difference was estimated using 200,000 within-group resamples with replacement (seed 20260811). Leave-one-out analysis repeated the group mean difference after removing each sample and recorded direction reversals. These analyses were intended as stress tests of the PHN-associated IFN result rather than as independent validation.

The expanded PHN-suggestive trend gene sets used for exploratory downstream prioritization were defined *post hoc* and are not treated as formal differential-expression signatures. The UP set was the union of leading-edge genes from significantly positively enriched Hallmark pathways and genes with PHN-versus-HZ log2FoldChange > 1 with nominal p < 0.05; the DOWN set was defined analogously using significantly negatively enriched Hallmark leading edges and log2FoldChange < -1 with nominal p < 0.05. This rule yielded 366 UP and 159 DOWN genes and was used only for exploratory regulatory/pathway prioritization.

### 2.5 Immune score estimation

Single-sample GSEA (ssGSEA) was performed using GSVA (Hanzelmann et al., 2013) with five literature-based immune signatures: IFN (IFI27, IFI44L, ISG15, RSAD2, IRF7, IFIT1, MX1, OAS1, OAS2, IFIT3, HERC5, DDX58, IFI6, EPSTI1), Cytotoxicity (GZMA, GZMB, GZMK, GZMH, PRF1, NKG7, GNLY, FGFBP2, SPON2, KLRD1, KLRF1, KLRG1), Inflammatory Monocyte (S100A8, S100A9, S100A12, IL1B, TNF, FCN1, VCAN, CD14, FCGR3A, LYZ, S100A6, ANXA1), Chemokine (CCL2, CCL3, CCL4, CCL5, CXCL8, CXCL9, CXCL10, CXCL11, CXCL13, CCR1, CCR2, CXCR3, CCR5, CCL19, CCL20), and Pain/Neuroinflammation (NGF, BDNF, CXCL13, IL6, TNF, IL1B, CCL2, TGFB1, IL10, VEGFA, CCL3, CCL5, NRXN3, SCN10A).

### 2.6 Cell-type deconvolution

Computational deconvolution was performed using xCell (Aran et al., 2017) (64 immune and stromal cell types) and MCP-counter (Becht et al., 2016) (10 cell populations). Statistical significance between groups was assessed using the Wilcoxon rank-sum test with Benjamini-Hochberg correction.

### 2.7 WGCNA

Weighted Gene Co-expression Network Analysis (WGCNA) (Langfelder and Horvath, 2008) was performed on GSE242252 (n=77, excluding ERC samples) using the top 5,000 most variable genes by median absolute deviation. A soft threshold power of 18 was selected to achieve approximate scale-free topology (R2 > 0.85). Modules were identified using dynamic tree cutting (minModuleSize=30, mergeCutHeight=0.25) and correlated with clinical traits (group, immune scores, cell-type proportions). Hub genes were defined as genes with module membership (kME) > 0.7 and gene-trait significance > 0.2. WGCNA results were interpreted within GSE242252 only and were not projected onto PRJNA1142765.

### 2.8 NicheNet ligand-target regulatory inference from bulk transcriptomic signatures

NicheNet (Browaeys et al., 2019) was used to infer ligand-target regulatory links using the human ligand-receptor network, ligand-target matrix, and weighted networks (Zenodo, December 2021 release). Upregulated gene sets served as the genes of interest, with all expressed genes as background. The top 20 ligands by area under the precision-recall curve (AUPR) were identified, and ligand-target links with weight > 0.01 were retained. Because the available datasets were bulk RNA-seq, NicheNet results were interpreted strictly as ligand-target regulatory inference from bulk transcriptomic signatures rather than direct intercellular interactions or sender-receiver relationships at single-cell resolution.

### 2.9 Transcription factor activity

Transcription factor (TF) activities were estimated using DoRothEA regulons (Garcia-Alonso et al., 2019) with the VIPER algorithm implemented in decoupleR (Badia-i-Mompel et al., 2022). High-confidence regulons (confidence levels A and B) were used. TF activity differences were calculated between groups and compared across datasets.

### 2.10 Pathway-oriented druggable-axis prioritization

Pathway-oriented druggable-axis prioritization was performed using two evidence layers: (1) druggable target and pharmacological-class annotation using curated DrugBank (Knox et al., 2024) knowledge for selected immune genes; and (2) Open Targets (Ochoa et al., 2021) annotation of target-disease associations for herpes zoster (MONDO_0005609), postherpetic neuralgia (MONDO_0041052), neuralgia (MONDO_0021667), and viral infection (MONDO_0005108). Prioritization was intentionally restricted to IFN-stimulated antiviral nodes (ISG15, RSAD2, USP18), STAT2, IFNG/IL27, and CCL5/CCR5. Broad inflammatory cytokine and immune-checkpoint axes, including TNF, IL6, PDCD1, and CD274, were treated as background immune-context signals rather than prioritized druggable axes because of their higher translational and safety ambiguity in infection-associated conditions. Open Targets evidence was considered database-level annotation rather than experimental or clinical validation.

### 2.11 Literature-based concordance and external replication feasibility search

Literature-based concordance was assessed using VZV IFN-defense literature (Sen et al., 2018; Gerada et al., 2020), HZ PBMC single-cell evidence (Zheng et al., 2024), PHN/HZ immune profiling (Peng et al., 2022), IFN-regulatory literature (Nowicka et al., 2023), neuroimmune context literature (Barragan-Iglesias et al., 2020; Tan et al., 2021; Franco-Enzastiga et al., 2025), antiviral-gene literature (Basters et al., 2018; Perng and Lenschow, 2018; Ghosh and Marsh, 2020), herpetic-neuralgia chemokine literature (Wu et al., 2023), and targeted serum IFN-subtype profiling (Chen et al., 2021). These sources were used only as supportive biological context and were not labeled as independent validation. In addition, a structured external-replication feasibility search was conducted. GEO was searched using seven structured queries (182 unique records; 38 retained as potentially relevant), ENA using 11 field-specific queries, BioStudies/ArrayExpress-associated records were screened (n=200), and 21 relevant publications were reviewed for additional datasets. Candidate datasets were assessed for species, tissue, design, group sizes, PHN definition/timing, platform, public sample-level availability, independence from PRJNA1142765, and suitability for direct replication. PRJNA1142765 and its source publication were excluded because they involve the same participants as the discovery cohort; GSE242252 was excluded from external-replication status because it was already part of the present analysis and contains no PHN samples. The complete inventory and inclusion/exclusion rationale are provided in **Supplementary Table 4**.

## 3 Results

Results are presented in an evidence-graded sequence: acute HZ activation and recovery, PHN-versus-HZ pathway robustness, cross-cohort directional context, regulatory inference, external-replication feasibility, and restricted candidate-axis prioritization. This order separates the strongest within-cohort evidence from exploratory and supportive analysis.

### 3.1 HZ onset shows type I IFN-dominated immune activation

Differential expression analysis of HZ onset versus all controls (n=28) identified 37 upregulated genes and 0 downregulated genes at |log2FC| >= 1 and padj < 0.05. The upregulated genes included IFN/antiviral genes (IFI27, log2FC=2.69; ISG15, 1.74; RSAD2, 1.87; HERC5, 1.19), cell cycle genes (CDK1, 1.76; BIRC5, 1.74; AURKB, 1.01; CDC20, 1.22; CEP55, 1.12), complement component C4BPA (2.04), and immunoglobulin genes (IGHG1, 1.46; IGHG4, 1.97; IGKV4-1, 1.49).

GSEA revealed significant enrichment of HALLMARK_INTERFERON_ALPHA_RESPONSE (NES = 2.21, padj=4.4x10-10), HALLMARK_INTERFERON_GAMMA_RESPONSE (NES = 2.05, padj=6.0x10-11), HALLMARK_E2F_TARGETS (NES = 2.29, padj=1.7x10-18), and HALLMARK_G2M_CHECKPOINT (NES = 2.21, padj=1.1x10-16). HALLMARK_HEME_METABOLISM was significantly downregulated (NES=-2.84, padj=1.2x10-20) (**Figure 2**).

**Figure 2:**
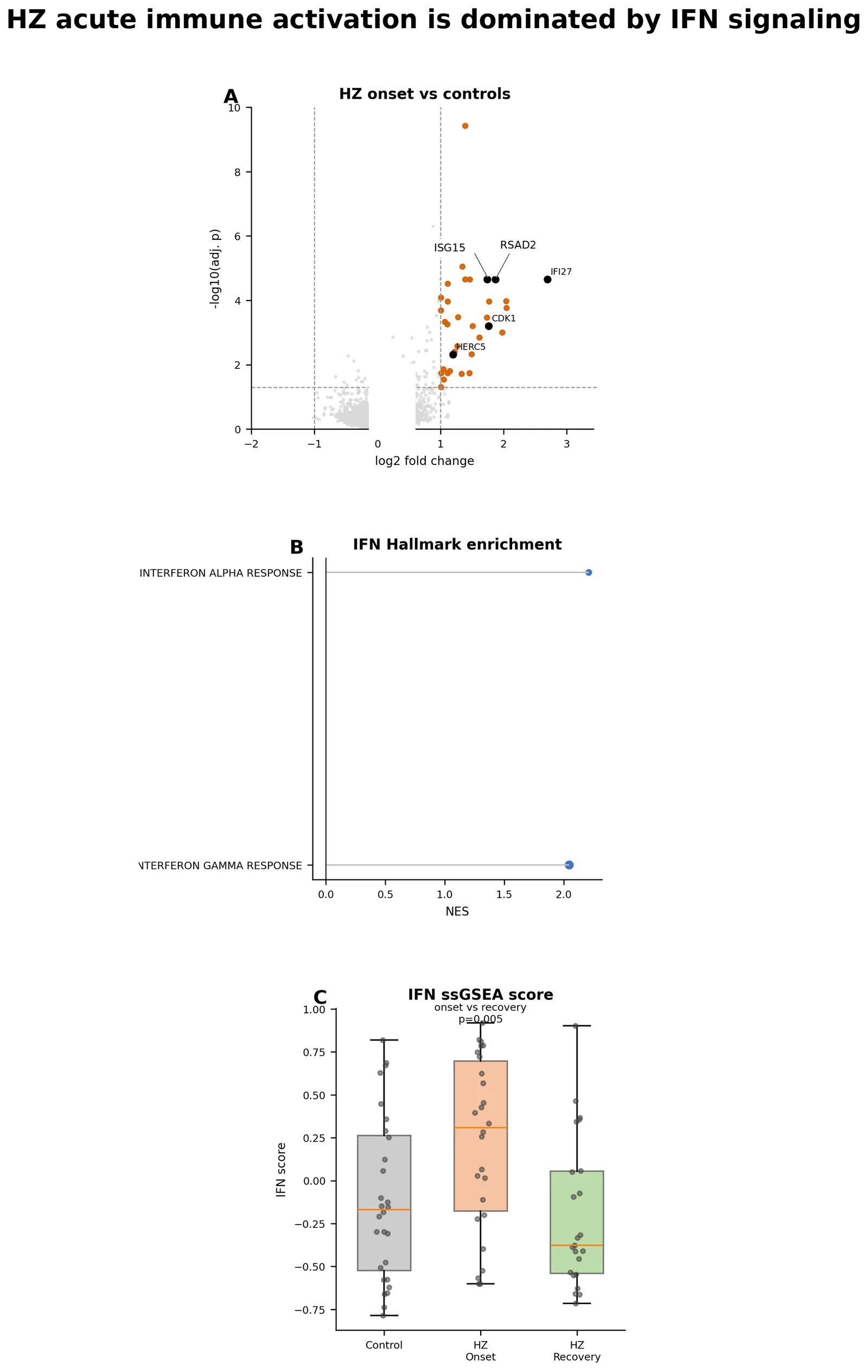
HZ acute immune activation is dominated by IFN signaling. **(A)** Volcano plot of HZ onset versus controls. **(B)** Hallmark IFN-alpha and IFN-gamma enrichment in HZ onset. **(C)** IFN ssGSEA score across control, HZ onset, and recovery groups.

Cell-type deconvolution using xCell identified FDR-significant increases in plasma cells (p=0.004, FDR = 0.026) and Th2 cells (p=0.001, FDR = 0.012) in HZ onset versus controls; macrophages were nominally higher (p=0.042). The IFN ssGSEA score was higher in HZ onset versus controls (p=0.016) and HZ onset versus recovery (p=0.005), while the chemokine score differed between onset and recovery (p=0.027) (**Figure 3**).

**Figure 3:**
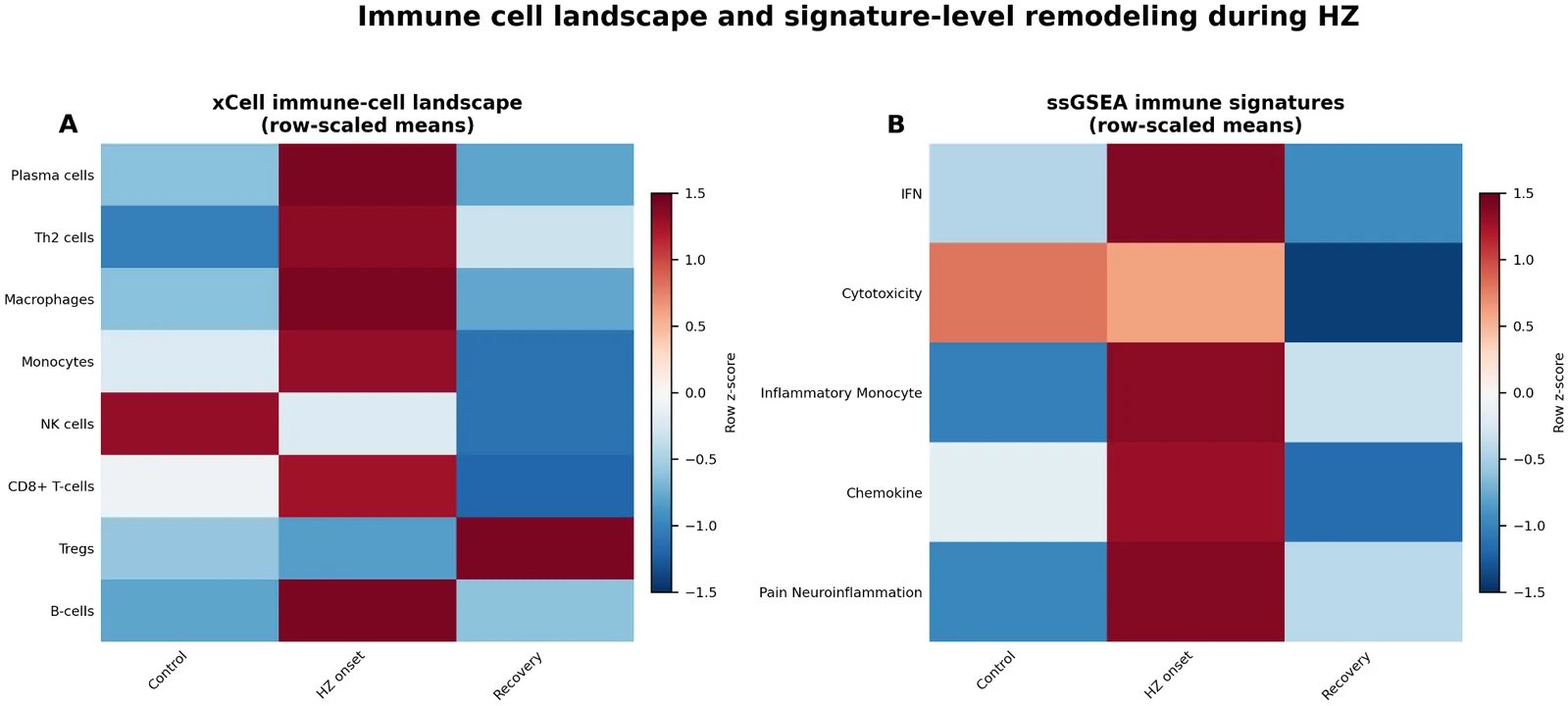
Immune cell landscape and signature-level remodeling during HZ. **(A)** xCell-derived immune-cell landscape across control, HZ onset, and recovery groups. **(B)** ssGSEA immune-signature heatmap showing IFN, cytotoxicity, inflammatory monocyte, chemokine, and pain/neuroinflammation signatures.

### 3.2 WGCNA identifies an IFN-enriched co-expression module

WGCNA on GSE242252 (n=77) identified 18 co-expression modules from 5,000 genes. Four modules showed nominal associations with HZ onset (p < 0.05). The MEred module was among these and was enriched for IFN/antiviral genes, with hub genes including STAT1 (kME=0.92), RSAD2 (0.92), GBP1 (0.93), IFIH1 (0.93), UBE2L6 (0.92), PARP9 (0.92), and CMPK2 (0.91). Gene Ontology enrichment of the MEred module yielded 392 Biological Process terms, predominantly related to type I IFN response, antiviral defense, and immune regulation (**Figure 4**).

**Figure 4:**
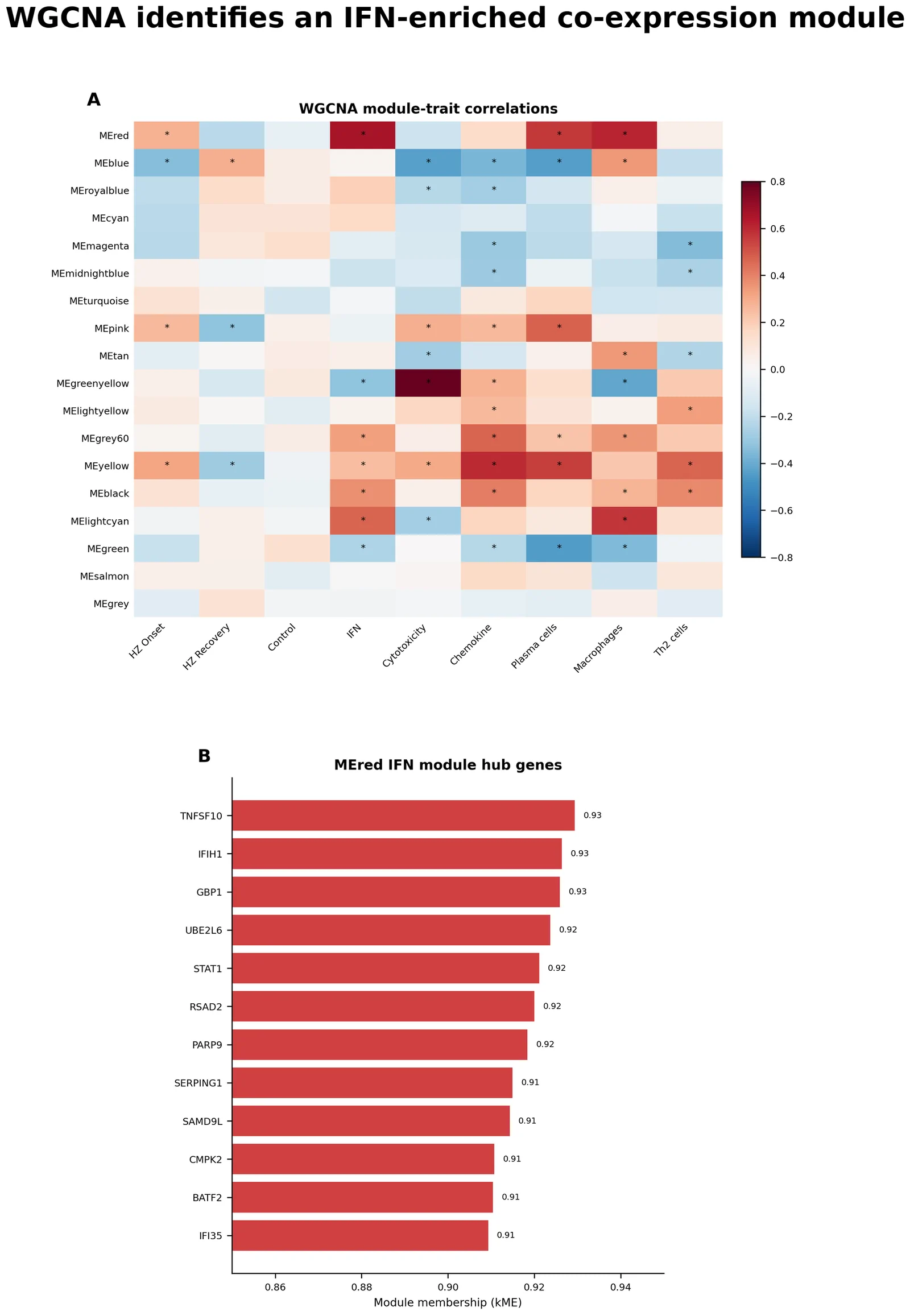
WGCNA identifies an IFN-enriched co-expression module. **(A)** Module-trait heatmap showing correlations between WGCNA modules and clinical or immune features. The asterisks (*) denote nominally significant module -trait associations at p < 0.05. **(B)** Top hub genes in the IFN-enriched MEred module.

### 3.3 PHN-versus-HZ differences are predominantly pathway-level rather than single-gene

In PRJNA1142765 (8 PHN vs. 15 HZ), only 1 annotated gene (HBG1, log2FC=5.52) was significantly upregulated and 1 unannotated gene was downregulated at the strict threshold, indicating limited individual-gene power in this small cohort. The corrected Hallmark GSEA (minSize=10, maxSize=500) showed positive enrichment of HALLMARK_INTERFERON_ALPHA_RESPONSE (NES = 2.572, FDR = 5.84x10-14), HALLMARK_INTERFERON_GAMMA_RESPONSE (NES = 2.178, FDR = 1.36x10-10), HALLMARK_HEME_METABOLISM (NES = 2.090, FDR = 2.08x10-9), and HALLMARK_OXIDATIVE_PHOSPHORYLATION (NES = 1.810, FDR = 6.04x10-5), whereas HALLMARK_EPITHELIAL_MESENCHYMAL_TRANSITION was negatively enriched (NES=-1.657, FDR = 0.0150). These findings were interpreted at the pathway level rather than as biomarker discovery.

Ranking-sensitivity analysis supported the stability of the IFN pathway ordering. With log2FoldChange ranking, IFN-alpha remained rank 1/50 (NES = 2.572, FDR = 5.84x10-14) and IFN-gamma rank 2/50 (NES = 2.178, FDR = 1.36x10-10). With Wald-statistic ranking, IFN-alpha remained rank 1/50 (NES = 2.755, FDR = 1.10x10-15) and IFN-gamma rank 2/50 (NES = 2.271, FDR = 2.13x10-12). Leading-edge overlap between rankings was high (Jaccard=0.848 for IFN-alpha and 0.897 for IFN-gamma). At the sample-score level, the observed PHN-minus-HZ mean IFN-score difference was 0.240 (PHN mean 0.107; HZ mean -0.133), with a 95% percentile bootstrap CI of -0.217 to 0.687; the exact patient-label permutation was not significant (two-sided p=0.297). Across all 23 leave-one-out iterations, the PHN-minus-HZ difference remained positive (range 0.120-0.361; 0 direction reversals). Taken together, the PHN-associated IFN signal was classified as partially robust: stable at the pathway-ranking level and directionally stable to sample deletion, but statistically imprecise at the patient-score level.

Key IFN genes showed directional fold-change increases in PHN versus HZ despite not reaching individual-gene significance, including IFI27 (log2FC=3.37), IFI44L (2.65), ISG15 (2.29), RSAD2 (2.16), IRF7 (1.65), MX1 (1.43), and IFIT1 (1.43). The cytotoxicity score showed a nonsignificant trend (p=0.087). The exploratory expanded PHN-suggestive sets comprised 366 UP and 159 DOWN genes according to the *post hoc* exploratory reconstruction rule described in Methods and were used only for downstream hypothesis generation.

### 3.4 Parallel cohort comparison shows directional IFN-related concordance

Within their respective cohorts, the IFN score was higher in HZ onset versus controls and directionally higher in PHN versus HZ. Plasma-cell and macrophage estimates were also directionally higher in both comparisons. Because the datasets were not pooled and the biological contrasts were confounded with study-specific technical features, these observations are presented as descriptive cross-cohort directional concordance rather than as a formally integrated or meta-analytic effect.

Transcription factor activity analysis using DoRothEA/decoupleR identified STAT2 (onset vs. control: +0.64; PHN vs. HZ: +0.63), IRF9 (+0.55; +0.50), and IRF1 (+0.28; +0.27) as directionally concordant within-cohort activity estimates. These factors are components of IFN-related regulatory programs, but the cross-cohort comparison does not establish a shared causal mechanism or temporal continuity (**Figure 5**).

**Figure 5:**
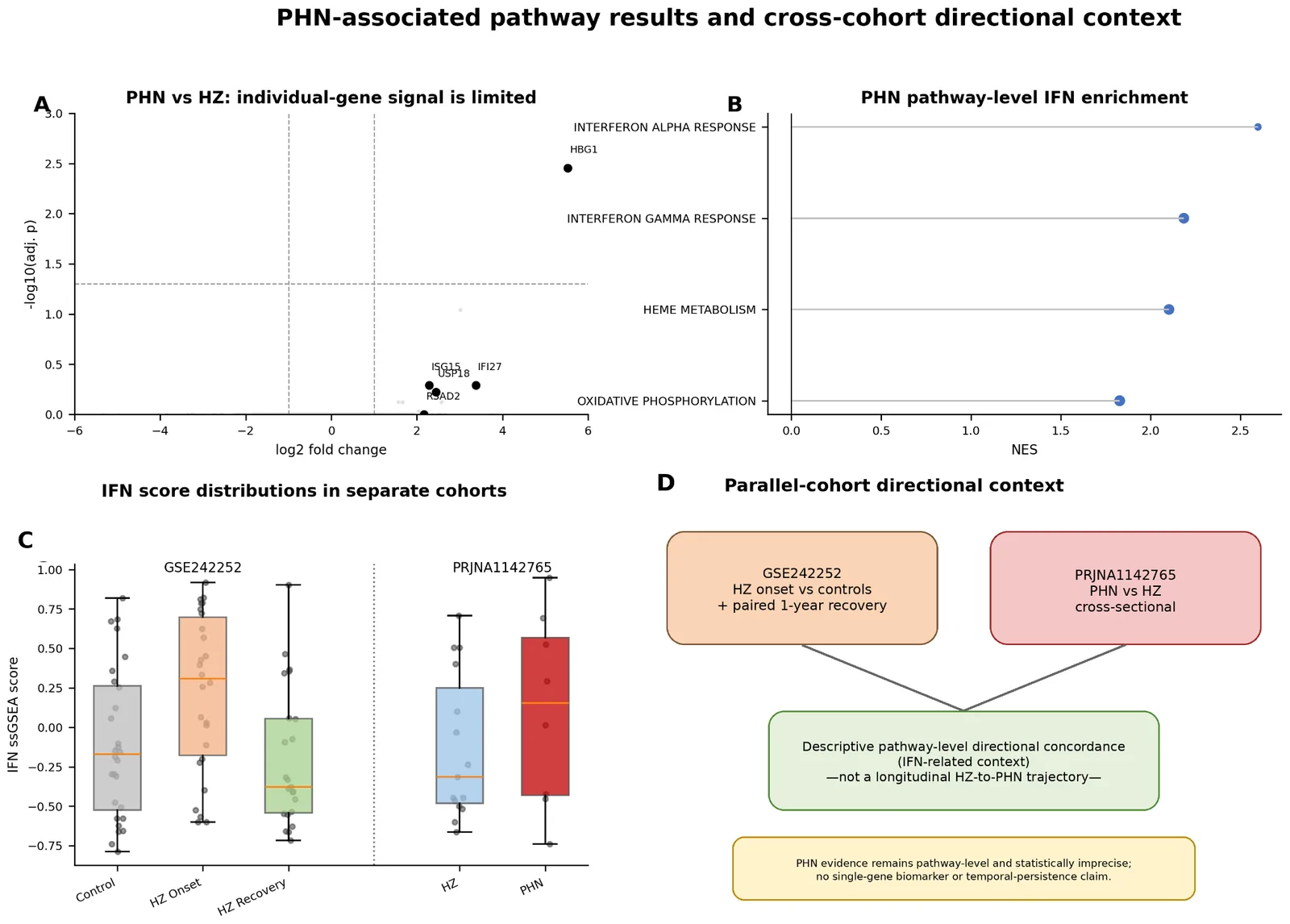
PHN-associated pathway results and cross-cohort directional context. **(A)** PHN versus HZ volcano plot showing limited individual-gene significance. **(B)** Hallmark enrichment in PHN versus HZ. **(C)** IFN-score distributions shown side-by-side for the two separate cohorts; values are not pooled and do not represent a common longitudinal scale. For the PRJNA1142765 PHN-versus-HZ contrast, the observed mean IFN-score difference was 0.240 (95% percentile bootstrap CI, -0.217 to 0.687; exact two-sided label-permutation p=0.297). **(D)** Schematic showing two independent analytical branches: HZ onset/recovery and PHN-versus-HZ. The branches converge only at descriptive pathway-level directional concordance and do not represent a longitudinal HZ-to-PHN trajectory.

### 3.5 Bulk-derived ligand-target inference highlights candidate regulatory axes in the PHN-versus-HZ comparison

NicheNet ligand-target regulatory inference from bulk transcriptomic signatures for HZ onset versus controls identified 45 active ligand-target links, with top predicted ligands including CXCL1, FGF2, ANGPTL4, and FCGR3B. In PHN versus HZ, 327 active ligand-target links were identified, with top predicted ligands including IL27 (AUPR_corrected=0.058), IFNG (0.039), IFITM1 (0.029), IL1B (0.020), HLA-DRB5 (0.020), CD226 (0.024), BST2 (0.023), and TNFSF4/OX40L (0.016). The PHN-suggestive ligand-target landscape highlighted IFN-related, inflammatory, and antigen-presentation-related regulatory axes.

### 3.6 Literature-based concordance and external replication search

A structured multi-database search did not identify a suitable independent publicly accessible human blood/PBMC transcriptomic cohort containing PHN samples that could directly replicate the PHN-versus-HZ comparison. PRJNA1142765 remained the only directly comparable public PHN-versus-HZ blood transcriptomic cohort identified. HRA008316 (Zheng et al., 2024) was the strongest independent partial candidate, but it contains HZ onset, recovery, and healthy PBMC samples without PHN and therefore cannot test the core PHN-versus-HZ finding. Other identified datasets involved different tissues, experimental VZV models, vaccine studies, or non-transcriptomic assays and were treated only as supportive biological context. Accordingly, the manuscript uses the terms literature-based concordance and supportive external context rather than external validation. The full search inventory is provided in **Supplementary Table 4**.

### 3.7 Restricted computational prioritization identifies IFN- and CCR5-related candidate axes

Pathway-oriented druggable-axis prioritization was narrowed to immunologically plausible and less overextended axes: ISG15, RSAD2, USP18, STAT2, IFNG/IL27, and CCL5/CCR5. ISG15, RSAD2, and USP18 appeared across HZ onset DEGs, the expanded PHN-suggestive trend gene set, and WGCNA hub genes. STAT2 was prioritized as a regulatory node because of its consistent TF activity trend and IFN-pathway position. IFNG/IL27 were retained as cytokine regulatory axes from the PHN-suggestive ligand-target inference layer. CCL5/CCR5 was retained as a chemokine axis because of external herpetic neuralgia literature and Open Targets viral-infection annotation. Broad inflammatory cytokine or immune-checkpoint axes, including TNF, IL6, PDCD1, and CD274, were not carried forward as prioritized druggable axes in the main text because they are more clinically ambiguous in infection-associated settings (**Figure 6**; **Supplementary Figure 10**).

**Figure 6:**
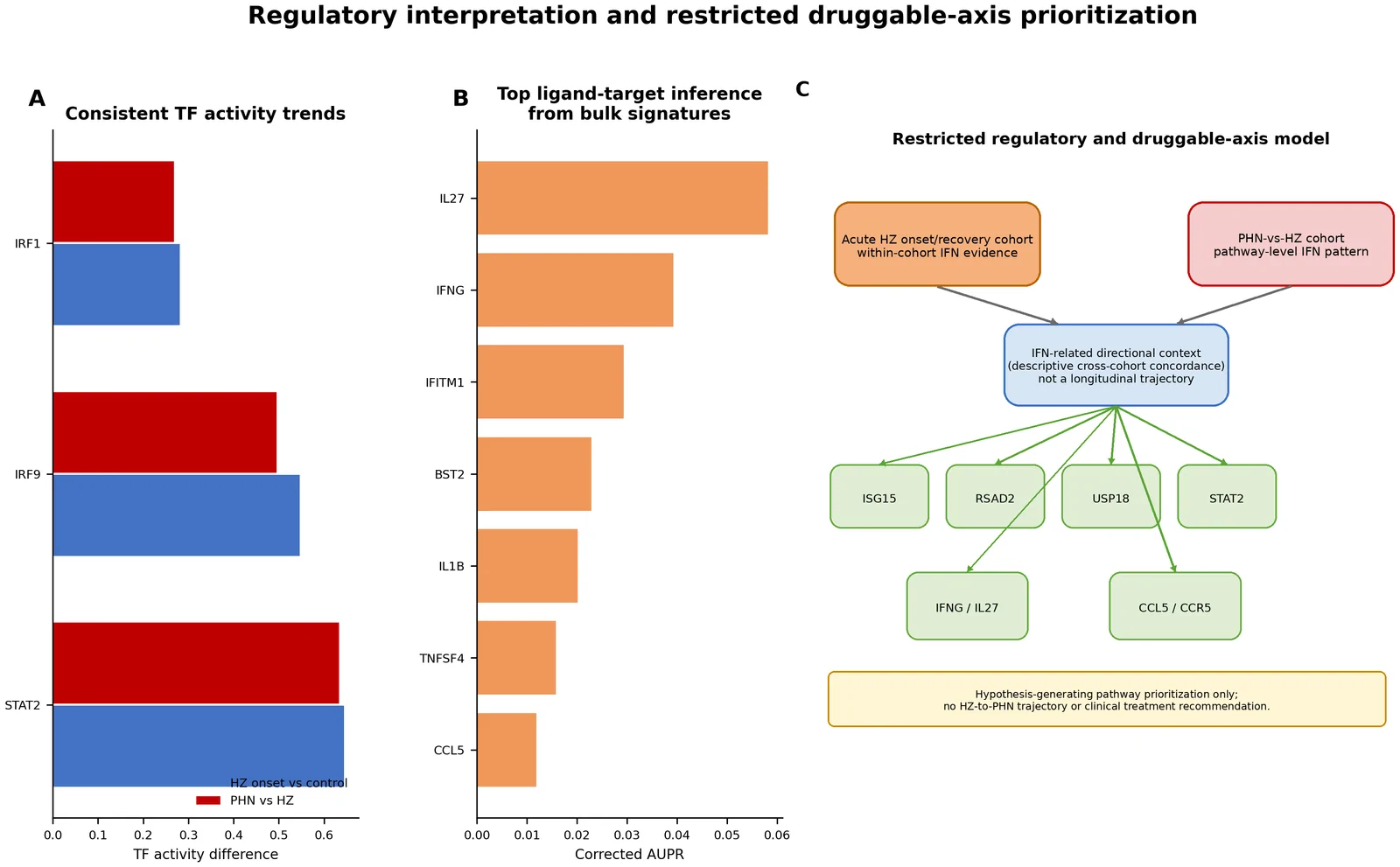
Regulatory interpretation and restricted druggable-axis prioritization. **(A)** Directionally consistent STAT2, IRF9, and IRF1 activity trends. **(B)** Top ligand-target regulatory inference from bulk transcriptomic signatures. **(C)** Restricted model in which the acute-HZ onset/recovery cohort and the separate PHN-versus-HZ cohort converge only at descriptive IFN-related directional context before hypothesis-generating prioritization of selected candidate axes. The schematic does not represent a longitudinal HZ-to-PHN trajectory or a clinical treatment recommendation.

Open Targets annotation was used only as database-level context. CCR5 showed a strong association with viral infection, and selected IFN-related nodes had viral-infection or immune-pathway annotations. However, these data were not interpreted as therapeutic validation. The narrowed prioritization emphasizes pathway plausibility and future experimental tractability rather than immediate clinical intervention. Detailed axis-level evidence is provided in **Supplementary Figure 10** and **Supplementary Table 1**.

## 4 Discussion

This study presents parallel analysis of two independent blood transcriptomic cohorts addressing different stages and contrasts of VZV-associated disease. The strongest evidence concerns acute HZ, where IFN-related activation was observed across differential-expression, pathway, immune-score, and co-expression analysis and attenuated during paired recovery. In contrast, the PHN-versus-HZ cohort yielded little single-gene significance and a pathway-level IFN signal that remained highly ranked under alternative ranking metrics but was not significant by exact permutation of the sample-level IFN score. The new biological information is therefore the asymmetry of evidence: acute HZ IFN activation and recovery-associated attenuation are robust, whereas the PHN-associated IFN pattern is stable at the pathway-ranking level but statistically uncertain at the patient level. Consequently, the earlier interpretation of persistent IFN activation in PHN should be narrowed rather than reinforced.

### 4.1 Evidence hierarchy and biological contribution

The principal biological advance over the 2024 HZ/PHN report (Wang et al., 2024) is not the number of computational methods applied, but the revised biological judgment produced by explicit evidence separation. The independent onset/recovery cohort supports strong acute-HZ IFN activation and recovery-associated attenuation, whereas the PHN-versus-HZ cohort is analyzed separately and stress-tested for small-sample robustness. The PHN IFN signal remains stable in pathway rank and leading-edge composition but is statistically uncertain at the patient-score level. Thus, the present analysis does not simply re-report an IFN signal; it shows that the strength of evidence is asymmetric and that the previous PHN persistence interpretation requires narrowing rather than reinforcement.

### 4.2 Acute HZ IFN activation and recovery

The dominant finding of type I IFN activation in HZ onset is consistent with the established antiviral role of IFN-alpha/beta and IFN-gamma responses to VZV (Sen et al., 2018; Gerada et al., 2020). HZ-onset GSEA enriched both IFN-alpha and IFN-gamma Hallmarks, and WGCNA identified an IFN-enriched module containing STAT1, RSAD2, GBP1, IFIH1, UBE2L6, PARP9, and CMPK2. Directionally concordant STAT2, IRF9, and IRF1 activity estimates across the two independent comparisons provide additional pathway-level context (Nowicka et al., 2023), but they should not be interpreted as evidence that the same regulatory state persists longitudinally from acute HZ into PHN.

### 4.3 PHN-associated IFN pattern and robustness

The PHN result requires a more constrained interpretation. Although IFN-alpha and IFN-gamma remained the top two positively enriched Hallmark pathways under both log2FoldChange and Wald-statistic rankings, the exact patient-label permutation test for the sample-level IFN score was not significant, and the 95% bootstrap confidence interval for the mean group difference included zero (-0.217 to 0.687). The absence of direction reversals in leave-one-out analysis suggests that the observed group direction was not driven by a single sample, but it does not overcome the limited n=8 PHN sample size. We therefore describe the finding as a PHN-associated peripheral-blood pathway pattern with statistical uncertainty, not as persistent IFN activation, a prognostic biomarker, or evidence that IFN signaling contributes causally to PHN pathogenesis.

### 4.4 Alternative biological context beyond IFN

The PHN-versus-HZ GSEA also identified non-IFN pathways, including positive heme-metabolism and oxidative-phosphorylation enrichment and negative epithelial-mesenchymal-transition enrichment. These signals raise alternative possibilities involving erythroid composition, systemic metabolic remodeling, or broader inflammatory-state differences. In parallel, established PHN biology includes neuroinflammation, neuronal injury, and sensory-neural remodeling (Li et al., 2024; Liu et al., 2024). The present whole-blood data cannot determine whether these neural processes are present, nor can they link peripheral IFN-related transcription directly to dorsal root ganglia or nociceptor biology. Neural and tissue-level mechanisms are therefore discussed only as external biological context.

### 4.5 Regulatory and candidate-axis interpretation

ISG15, RSAD2/viperin, USP18, and STAT2 emerged as recurrent computationally prioritized IFN-axis nodes. Their recurrence across acute-HZ differential expression, PHN exploratory gene sets, co-expression results, and IFN-related literature supports their use as hypotheses for future validation. It does not establish them as PHN biomarkers, mechanistic mediators, or therapeutic targets.

NicheNet ligand-target regulatory inference highlighted IL27, IFNG, IL1B, and antigen-presentation-related ligands in PHN. Because these analyses were performed on bulk RNA-seq data, the results cannot establish direct cell-cell communication or identify sender and receiver cell populations with single-cell resolution. They nevertheless provide a regulatory-prioritization layer that is consistent with IFN-related and inflammatory pathway trends. The CCL5/CCR5 axis, previously implicated in herpetic neuralgia (Wu et al., 2023), was retained as a narrowed candidate chemokine axis because it combines bulk-derived directionality, external literature context, and database-level viral-infection annotation.

The druggable-axis prioritization analysis should be interpreted cautiously and was intentionally restricted in the present analysis. The main text prioritizes only ISG15, RSAD2, USP18, STAT2, IFNG/IL27, and CCL5/CCR5. Broad inflammatory cytokine and immune-checkpoint axes, including TNF, IL6, PDCD1, and CD274, were not carried forward as main druggable axes because they are more clinically ambiguous in infection-associated conditions and could invite overinterpretation as immunotherapy recommendations. The appropriate interpretation is that the retained axes define pathways and nodes that merit experimental validation, not immediate prevention or treatment strategies for HZ or PHN.

### 4.6 Limitations

Several limitations define the boundary of the present study. First, PHN is a peripheral nervous system pain disorder, whereas the analyzed human data are from whole blood. Peripheral-blood transcription cannot directly characterize dorsal root ganglia, sensory neurons, satellite glial cells, or local neural-immune interactions; consequently, no neural mechanism or blood-to-neuron causal pathway is claimed. Second, GSE242252 and PRJNA1142765 differ in sequencing platform, library preparation, cohort design, processing, and clinical sampling structure. Because study identity is confounded with these technical and biological differences, a global batch correction or single quantitative batch-effect estimate would itself mix technical variation with differences in biological contrast. The datasets were therefore analyzed in parallel rather than merged, batch-corrected, or treated as a formal meta-analysis. Cross-cohort comparisons are limited to directional concordance of within-cohort summaries; the heterogeneity assessment and mitigation strategy are summarized in **Supplementary Table 5**.

Third, the PHN cohort is small (8 PHN and 15 HZ), resulting in minimal strict single-gene significance and limited patient-level precision despite stable pathway rankings. Available source metadata showed similar average age, VAS, sex distribution, zoster location, and chronic-disease distributions, but detailed antiviral regimens, vaccination status, analgesic exposure, and exact participant-level sampling intervals were unavailable and could not be adjusted for. Fourth, the cross-sectional PHN-versus-HZ comparison cannot establish temporal persistence from acute HZ to PHN. Fifth, the structured replication search did not identify a suitable independent public PHN blood/PBMC transcriptomic cohort, so the PHN-associated result remains unreplicated. The feasibility search was structured but was not a formal systematic review and did not comprehensively query every regional or non-English repository. Sixth, bulk-derived NicheNet, TF activity estimates, and druggable-axis annotations are computational inferences and remain hypothesis-generating.

Within these constraints, the study has three main strengths. First, it provides a well-supported within-cohort view of acute HZ and recovery. Second, it stress-tests the small PHN cohort rather than relying on pathway FDR alone. Third, it explicitly separates direct evidence, exploratory analysis, supportive literature, and unavailable validation. This evidence-graded framework improves interpretability and reproducibility without implying mechanistic certainty.

## 5 Conclusion

This study supports IFN-dominated peripheral-blood immune activation as a major feature of acute HZ and demonstrates attenuation during recovery in an independent longitudinal HZ cohort. In the separate PHN-versus-HZ cohort, IFN-alpha and IFN-gamma pathways remained highly ranked across sensitivity analysis, whereas patient-level IFN-score permutation was not significant. The PHN result should therefore be regarded as a partially robust but statistically imprecise pathway-level pattern that requires independent replication. The available data do not establish temporal persistence, neural mechanisms, causal progression to PHN, or validated therapeutic targets. The restricted IFN-related and CCL5/CCR5-associated nodes are best viewed as hypotheses for future experimental and prospective clinical evaluation.

## Data Availability

All datasets analyzed in this study are publicly available. The HZ onset and recovery dataset is available from the Gene Expression Omnibus under accession number GSE242252, and the HZ/PHN RNA-seq dataset is available from the Sequence Read Archive under accession number PRJNA1142765. Other public resources used in the analysis include MSigDB, DrugBank, and Open Targets. **Supplementary Tables S1–S5** provide the processed result tables used for the restricted candidate-axis summary, corrected Hallmark GSEA, PHN robustness analyses, external-replication feasibility assessment, and structured cohort-heterogeneity comparison. Additional derived analysis materials are available from the corresponding author upon reasonable request.

## Supplementary material

Supplementary Figure 1 Full parallel-analysis workflow. The expanded workflow shows cohort-specific acquisition and preprocessing, independent HZ onset/recovery and PHN-versus-HZ analysis, PHN robustness testing, descriptive cross-cohort pathway/regulatory concordance, the structured external-replication search, and restricted hypothesis-generating regulatory prioritization. The two human cohorts remain analytically separate throughout.

Supplementary Figure 2 Full volcano plots for HZ onset versus controls and PHN versus HZ.

Supplementary Figure 3 Full Hallmark GSEA summaries for HZ onset versus controls and PHN versus HZ.

Supplementary Figure 4 Full xCell immune-cell panels across control, HZ onset, and recovery groups.

Supplementary Figure 5 Full MCP-counter output across control, HZ onset, and recovery groups.

Supplementary Figure 6 Full WGCNA module-trait relationship heatmap across all modules and traits.

Supplementary Figure 7 Full NicheNet ligand-target regulatory heatmap for PHN versus HZ.

Supplementary Figure 8 Key ligand-receptor axis gene expression trends across HZ onset and PHN comparisons. This bulk-expression matrix summarizes log2 fold-change trends and should not be interpreted as direct cell-cell communication.

Supplementary Figure 9 External-replication search and evidence classification. The structured external-replication search covered GEO, ENA, BioStudies/ArrayExpress-associated records, and relevant publications. No suitable independent public human blood/PBMC transcriptomic cohort containing PHN samples was identified for direct replication of the PHN-versus-HZ IFN finding. HRA008316 was retained only as a Tier B partial external dataset because it contains HZ onset, recovery, and healthy PBMC samples but no PHN. Tier C and non-transcriptomic studies are presented only as supportive biological context, not independent validation.

Supplementary Figure 10 Restricted druggable-axis evidence matrix. This figure summarizes the narrowed IFN-related and CCR5-related axes retained for cautious pathway-oriented prioritization.

## References

[B1] Aran D. Hu Z. Butte A. J. (2017). xCell: digitally portraying the tissue cellular heterogeneity landscape. Genome Biol. 18, 220. doi: 10.1186/s13059-017-1349-1 29141660 PMC5688663

[B2] Badia-I-Mompel P. Vélez Santiago J. Braunger J. Geiss C. Dimitrov D. Müller-Dott S. . (2022). decoupleR: ensemble of computational methods to infer biological activities from omics data. Bioinf. Adv. 2, vbac016. doi: 10.1093/bioadv/vbac016 36699385 PMC9710656

[B3] Barragan-Iglesias P. Franco-Enzastiga U. Jeevakumar V. Shiers S. Wangzhou A. Granados-Soto V. . (2020). Type I interferons act directly on nociceptors to produce pain sensitization: implications for viral infection-induced pain. J. Neurosci. 40, 3517–3532. doi: 10.1523/JNEUROSCI.3055-19.2020 32245829 PMC7189756

[B4] Basters A. Knobeloch K. P. Fritz G. (2018). USP18 - a multifunctional component in the interferon response. Biosci. Rep. 38, BSR20180250. doi: 10.1042/bsr20180250 30126853 PMC6240716

[B5] Becht E. Giraldo N. A. Lacroix L. Buttard B. Elarouci N. Petitprez F. . (2016). Estimating the population abundance of tissue-infiltrating immune and stromal cell populations using gene expression. Genome Biol. 17, 218. doi: 10.1186/s13059-016-1070-5 27765066 PMC5073889

[B6] Boeren M. de Vrij N. Ha M. K. Valkiers S. Souquette A. Gielis S. . (2024). Lack of functional TCR-epitope interaction is associated with herpes zoster through reduced downstream T cell activation. Cell Rep. 43, 114062. doi: 10.1016/j.celrep.2024.114062 38588339

[B7] Browaeys R. Saelens W. Saeys Y. (2019). NicheNet: modeling intercellular communication by linking ligands to target genes. Nat. Methods 16, 179–188. doi: 10.1038/s41592-019-0667-5 31819264

[B8] Chen Y. C. Figliozzi R. W. Hsia S. V. (2021). Pilot analyses of interferon subtype expression profiles in patients with herpes zoster or postherpetic neuralgia. Viral Immunol. 34, 437–447. doi: 10.1089/vim.2020.0295 33857386 PMC8563454

[B9] Curran D. Callegaro A. Fahrbach K. Neupane B. Vroling H. van Oorschot D. . (2021). Meta-regression of herpes zoster incidence worldwide. Infect. Dis. Ther. 10, 2261–2273. doi: 10.1007/s40121-021-00567-8 34874546 PMC8650523

[B10] Franco-Enzastiga U. Natarajan K. Espinosa F. Granja-Vazquez R. Mydugolam H. Price T. J. (2025). Type I IFNs enhance human dorsal root ganglion nociceptor excitability and induce TRPV1 sensitization. JCI Insight 10, e194987. doi: 10.1172/jci.insight.194987 40892477 PMC12513479

[B11] Garcia-Alonso L. Holland C. H. Ibrahim M. M. Turei D. Saez-Rodriguez J. (2019). Benchmark and integration of resources for the estimation of human transcription factor activities. Genome Res. 29, 1363–1375. doi: 10.1101/gr.240663.118 31340985 PMC6673718

[B12] Gerada C. Campbell T. M. Kennedy J. J. McSharry B. P. Steain M. Slobedman B. . (2020). Manipulation of the innate immune response by varicella zoster virus. Front. Immunol. 10, 1. doi: 10.3389/fimmu.2020.00001 32038653 PMC6992605

[B13] Ghosh S. Marsh E. N. G. (2020). Viperin: an ancient radical SAM enzyme finds its place in modern cellular metabolism and innate immunity. J. Biol. Chem. 295, 17785–17795. doi: 10.1074/jbc.REV120.012784 32546482 PMC7450102

[B14] Hanzelmann S. Castelo R. Guinney J. (2013). GSVA: gene set variation analysis for microarray and RNA-Seq data. BMC Bioinf. 14, 7. doi: 10.1186/1471-2105-14-7 23323831 PMC3618321

[B15] Knox C. Wilson M. Klinger C. M. Franklin M. Oler E. Wilson A. . (2024). DrugBank 6.0: the drugBank knowledgebase for 2024. Nucleic Acids Res. 52, D1265–D1275. doi: 10.1093/nar/gkad976 37953279 PMC10767804

[B16] Laing K. J. Ouwendijk W. J. D. Koelle D. M. Verjans G. M. G. M. (2018). Immunobiology of varicella-zoster virus infection. J. Infect. Dis. 218, S68–S73. doi: 10.1093/infdis/jiy403 30247598 PMC6151075

[B17] Langfelder P. Horvath S. (2008). WGCNA: an R package for weighted correlation network analysis. BMC Bioinf. 9, 559. doi: 10.1186/1471-2105-9-559 19114008 PMC2631488

[B18] Li Y. Jin J. Kang X. Feng Z. (2024). Identifying and evaluating biological markers of postherpetic neuralgia: a comprehensive review. Pain Ther. 13, 1029–1047. doi: 10.1007/s40122-024-00640-3 39126594 PMC11393369

[B19] Liberzon A. Birger C. Thorvaldsdottir H. Ghandi M. Mesirov J. P. Tamayo P. (2015). The molecular signatures database hallmark gene set collection. Cell Syst. 1, 417–425. doi: 10.1016/j.cels.2015.12.004 26771021 PMC4707969

[B20] Liu Q. Han J. Zhang X. (2024). Peripheral and central pathogenesis of postherpetic neuralgia. Skin Res. Technol. 30, e13867. doi: 10.1111/srt.13867 39101621 PMC11299165

[B21] Love M. I. Huber W. Anders S. (2014). Moderated estimation of fold change and dispersion for RNA-seq data with DESeq2. Genome Biol. 15, 550. doi: 10.1186/s13059-014-0550-8 25516281 PMC4302049

[B22] Nowicka H. Sekrecka A. Blaszczyk K. Kluzek K. Chang C. Y. Wesoly J. . (2023). ISGF3 and STAT2/IRF9 control basal and IFN-induced transcription through genome-wide binding of phosphorylated and unphosphorylated complexes to common ISRE-containing ISGs. Int. J. Mol. Sci. 24, 17635. doi: 10.3390/ijms242417635 38139463 PMC10743977

[B23] Ochoa D. Hercules A. Carmona M. Suveges D. Gonzalez-Uriarte A. Malangone C. . (2021). Open Targets Platform: supporting systematic drug-target identification and prioritisation. Nucleic Acids Res. 49, D1302–D1310. doi: 10.1093/nar/gkaa1027 33196847 PMC7779013

[B24] Patil A. D. Goldust M. Wollina U. (2022). Herpes zoster: a review of clinical manifestations and management. Viruses 14, 192. doi: 10.3390/v14020192 35215786 PMC8876683

[B25] Patro R. Duggal G. Love M. I. Irizarry R. A. Kingsford C. (2017). Salmon: fast and bias-aware quantification of transcript expression using dual-phase inference. Nat. Methods 14, 417–419. doi: 10.1038/nmeth.4197 28263959 PMC5600148

[B26] Peng Q. Guo X. Luo Y. Wang G. Zhong L. Zhu J. . (2022). Dynamic immune landscape and VZV-specific T cell responses in patients with herpes zoster and postherpetic neuralgia. Front. Immunol. 13, 887892. doi: 10.3389/fimmu.2022.887892 35720399 PMC9199063

[B27] Perng Y. C. Lenschow D. J. (2018). ISG15 in antiviral immunity and beyond. Nat. Rev. Microbiol. 16, 423–433. doi: 10.1038/s41579-018-0020-5 29769653 PMC7097117

[B28] Sen N. Sung P. Panda A. Arvin A. M. (2018). Distinctive roles for type I and type II interferons and interferon regulatory factors in the host cell defense against varicella-zoster virus. J. Virol. 92, e01151-18. doi: 10.1128/jvi.01151-18 30089701 PMC6189497

[B29] Soneson C. Love M. I. Robinson M. D. (2015). Differential analyses for RNA-seq: transcript-level estimates improve gene-level inferences. F1000Research 4, 1521. doi: 10.12688/f1000research.7563.2 26925227 PMC4712774

[B30] Srivastava A. Malik L. Sarkar H. Zakeri M. Almodaresi F. Soneson C. . (2020). Alignment and mapping methodology influence transcript abundance estimation. Genome Biol. 21, 71. doi: 10.1186/s13059-020-02151-8 32894187 PMC7487471

[B31] Tan P. H. Ji J. Yeh C. C. Ji R. R. (2021). Interferons in pain and infections: emerging roles in neuro-immune and neuro-glial interactions. Front. Immunol. 12, 783725. doi: 10.3389/fimmu.2021.783725 34804074 PMC8602180

[B32] Vandoren R. Boeren M. Schippers J. Bartholomeus E. Mullan K. Michels N. . (2024). Unravelling the immune signature of herpes zoster: insights into pathophysiology and the HLA risk profile. J. Infect. Dis. 229, e128–e137. doi: 10.1093/infdis/jiad609 38195164 PMC11420803

[B33] Wang C. Zhang K. Bao Y. Liu Y. Zhou Y. Ji Y. H. . (2024). Exploring blood transcriptomic signatures in patients with herpes zoster and postherpetic neuralgia. Front. Cell. Infect. Microbiol. 14, 1425393. doi: 10.3389/fcimb.2024.1425393 39211798 PMC11358128

[B34] Wu T. Hu E. Xu S. Chen M. Guo P. Dai Z. . (2021). clusterProfiler 4.0: a universal enrichment tool for interpreting omics data. Innovation 2, 100141. doi: 10.1016/j.xinn.2021.100141 34557778 PMC8454663

[B35] Wu S. Yang S. Li R. Ba X. Jiang C. Xiong D. . (2023). HSV-1 infection-induced herpetic neuralgia involves a CCL5/CCR5-mediated inflammation mechanism. J. Med. Virol. 95, e28718. doi: 10.1002/jmv.28718 37185840

[B36] Zheng S. Zhang S. Li X. Fei Y. Yang L. Liu B. . (2024). Single-cell immune profiling and validation of PBMCs in the onset of and recovery from herpes zoster. Commun. Biol. 7, 1001. doi: 10.1038/s42003-024-07289-w 39633072 PMC11618686

